# Activation of renin-angiotensin system is involved in dyslipidemia-mediated renal injuries in apolipoprotein E knockout mice and HK-2 cells

**DOI:** 10.1186/1476-511X-12-49

**Published:** 2013-04-09

**Authors:** Jie Ni, Kun-Ling Ma, Chang-Xian Wang, Jing Liu, Yang Zhang, Lin-Li Lv, Hai-Feng Ni, Ya-Xi Chen, Xiong-Zhong Ruan, Bi-Cheng Liu

**Affiliations:** 1 Institute of Nephrology, Zhong Da Hospital, Southeast University School of Medicine, Nanjing City, Jiangsu Province, P.R. China; 2 Department of Infection management, Zhong Da Hospital, Southeast University School of Medicine, Nanjing City, Jiangsu Province, P.R. China; 3 Centre for Lipid Research, Key Laboratory of Metabolism on Lipid and Glucose, Chongqing Medical University, Chongqing, P.R. China; 4 Centre for Nephrology, University College London (UCL) Medical School, Royal Free Campus, London, UK

**Keywords:** Hyperlipidemia, Renin-angiotensin system, Epithelial-mesenchymal transition, Renal injury

## Abstract

**Background:**

Dyslipidemia and activation of renin-angiotensin system (RAS) contribute to the progression of chronic kidney disease (CKD). This study investigated possible synergistic effects of intrarenal RAS activation with hyperlipidemia in renal injuries.

**Methods:**

Apolipoprotein knockout mice were fed with normal chow diet (control) or high fat diet (HF group) for eight weeks. Human proximal tubular epithelial cell line (HK-2) was treated without (control) or with cholesterol (30 μg/ml) plus 25-hydroxycholesterol (1 μg/ml) (lipid group) for 24 hours. The plasma lipid profile and RAS components were determined by clinical biochemistry assay and radiommunoassay, respectively. Collagen deposition in kidneys was evaluated by Masson-staining. The gene and protein expressions of molecules involved in RAS components and biomarkers of epithelial mesenchymal transition (EMT) were examined by real-time PCR, immunochemical staining, and Western blot.

**Results:**

The mice fed with high-fat diet showed significant hyperlipidemia with collagen deposition in renal tubular interstitium compared to controls. The plasma levels of renin, angiotensin I, and angiotensin II were no difference in two groups. However, the kidneys of HF group showed up-regulated RAS components, which were positively associated with increased plasma levels of triglyceride, total cholesterol, and LDL. These effects were further confirmed by *in vitro* studies. Lipid loading induced HK-2 cells underwent EMT, which was closely associated with the increased expressions of intracellular RAS components.

**Conclusions:**

Local RAS activation was involved in hyperlipidemia-mediated renal injuries, suggesting that there are synergistic effects resulting from RAS activation with hyperlipidemia that accelerates the progression of CKD.

## Background

A growing body of evidence shows that injured renal tubular epithelial cells have been implicated in increasing kidney matrix-producing fibroblasts and myofibroblasts populations through the process of epithelial–mesenchymal transition (EMT), ultimately leading to renal tubular interstitial fibrosis (TIF) [1] and inevitable progressive chronic kidney disease (CKD) [2]. Dyslipidemia is one of the main risk factors for the progression of CKD [3]. CKD is mainly characterized by normal or low concentrations of low-density lipoprotein (LDL) cholesterol, increased concentrations of small dense LDL, very-low-density lipoprotein (VLDL), intermediate-density lipoprotein (IDL), and decreased concentrations of high-density lipoprotein cholesterol (HDL) [4]. Persistent impaired renal function in CKD patients is associated with a significant alteration in lipoprotein metabolism, depending on the decline in the glomerular filtration rate (GFR) and whether the CKD is accompanied by inflammatory stress [5]. Reduced clearance and increased plasma levels of small dense LDL particles facilitate their entrance into arterial walls, resulting in the overproduction of inflammatory mediators and reactive oxygen species, which is a major cause for LDL- mediated renal and vascular damage. At present, the exact mechanisms for dyslipidemia-mediated renal injuries have not been entirely elucidated.

In addition to dyslipidemia, the activation of the RAS has been implicated in the progression of CKD [6]. Angiotensin II is the most powerful biologically active product of RAS and activates at least two types of cell-surface receptors, type 1 receptor (AT1) and type 2 receptor (AT2). Angiotensin II directly constricts vascular smooth muscle cells, enhances myocardial contractility, stimulates aldosterone production, stimulates sodium and water retention, stimulates the release of catecholamines from the adrenal medulla and sympathetic nerve endings, and increases sympathetic nervous system activity, which results in increased blood pressure [6]. In addition, angiotensin II, via the AT1-dependent pathway, causes cell proliferation, production of pro-inflammatory mediators, and extracellular matrix synthesis, all of which facilitates kidney damage and accelerates the progression of CKD [7-9]. Recently, the focus of interest in the RAS has shifted toward the role of the local RAS in kidneys [10]. Locally produced angiotensin II induces inflammation, cell growth, cell migration, cell differentiation, and apoptosis. Angiotensin II also regulates gene expression of bioactive substances and activates multiple intracellular signaling pathways, all of which may contribute to renal injury [6]. Studies have shown that there is significant diversity in the mechanism of intracellular synthesis of RAS in various cell types and pathological conditions [11]. At present, the intracellular synthesis pathway of RAS in renal tubular cells and its stimuli remain unclear.

Increasing evidence reveals cross-talk between dyslipidemia and RAS activation in cardiovascular diseases [12,13]. Native or oxidized LDL, through LDL receptors and scavenger receptors, upregulates angiotensin-converting enzyme (ACE) and AT1 expression in human endothelial cells [14,15]. On the other hand, angiotensin II facilitates the oxidation of LDL and its uptake by smooth muscle cells and macrophages [16,17]. Although CKD is commonly accompanied by dyslipidemia and RAS activation, whether there is an interaction between these two factors in renal injuries is still unknown. This study was undertaken to investigate the role of RAS activation in hyperlipidemia-mediated renal injuries *in vivo* and *in vitro*.

## Results

### Hyperlipidemia was induced in HF group mice

As shown in Table 1, there were significantly increased plasma concentrations of TG, TC, and LDL in the HF group compared with the control group. There was no significant difference in body weights or in the ratio of kidney weight to body weight between the two groups.

**Table 1:** Biochemical data on the two groups of mice (mean ± SD)

**Indexes**	**Control group (n = 8)**	**HF group (n = 8)**
Body weight (g)	28.4 ± 2.0	28.2 ± 1.82
Kidney weight/body weight (mg/g)	14.0 ± 1.8	14.2 ± 1.5
TG (mmol/l)	3.1 ± 0.6	7.5 ± 2.0*
TC (mmol/l)	13.7 ± 2.7	27.8 ± 4.0*
HDL (mmol/l)	1.0 ± 0.4	0.8 ± 0.34
LDL (mmol/l)	5.8 ± 1.5	11.6 ± 3.0*

Compared to the controls, the mice fed with a high fat diet developed hyperlipidemia. TG: triglycerides, TC: total cholesterol, HDL: high-density lipoprotein, LDL: low-density lipoprotein. * *P* < 0.05 *vs.* control.

### Morphological changes of kidneys in two groups of mice

**Figure 1:**
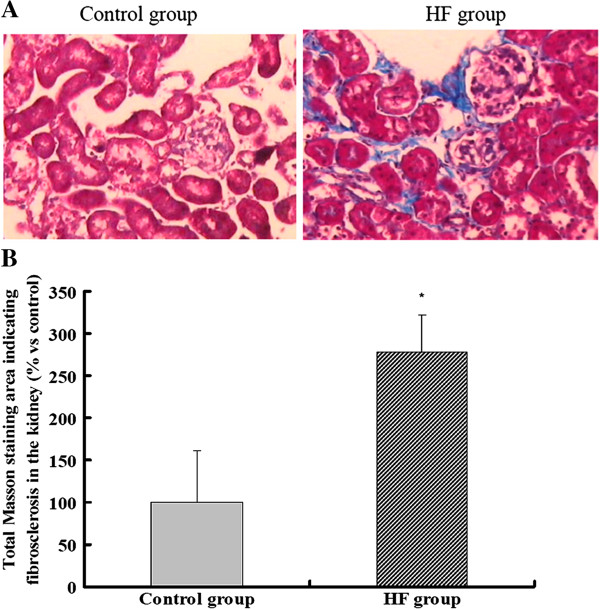
**Morphological changes in the kidneys of ApoE KO mice.** (**A**) Masson staining showed significant deposition of collagen in the tubular interstitium of the HF group when compared with the control group (×200). (**B**) Quantitative analysis of fibrotic tissue stained by Masson staining. Positive stainging was quantified by image analysis using Image J software by a point-counting technique under a 176-point grid. The histogram represents the mean ± SD of the percentage of the field area from eight experiments. * *P* < 0.05 *vs.* control.

We used Masson staining to evaluate hyperlipidemia-mediated renal pathological change in ApoE KO mice. The results showed that there was a significant accumulation of collagen fibers (stained blue) in the tubular interstitium in the HF group, which was further confirmed by quantitative analysis of positive staining using Image J software (Figure 1A and 1B).

### Intrarenal, not circulatory RAS, was activated in HF group mice

**Figure 2:**
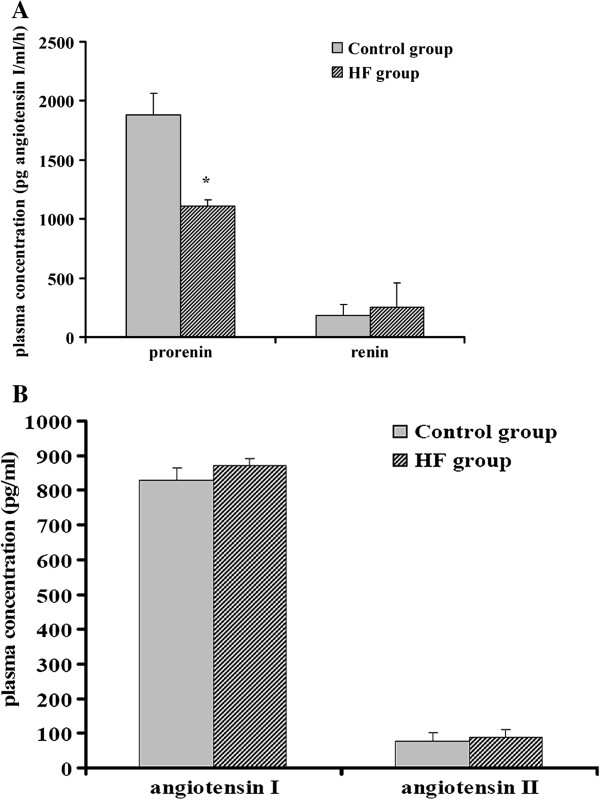
**Plasma concentrations of prorenin, renin, angiotensin I, and angiotensin II in the two groups of mice (A) and (B).** Plasma concentrations of renin, angiotensin I, and angiotensin II were not significant in the two groups although the plasma prorenin level was significantly reduced in the HF group. * *P* <0.05 *vs.* control.

To evaluate the circulating RAS levels in the two groups of ApoE KO mice, we measured plasma concentrations of prorenin, renin, angiotensin I, and angiotensin II. There was no difference in the plasma concentrations of renin, angiotensin I, and angiotensin II between the two groups; however, prorenin levels were significantly reduced in the HF group compared with the control group (Figure 2A and 2B).

**Figure 3:**
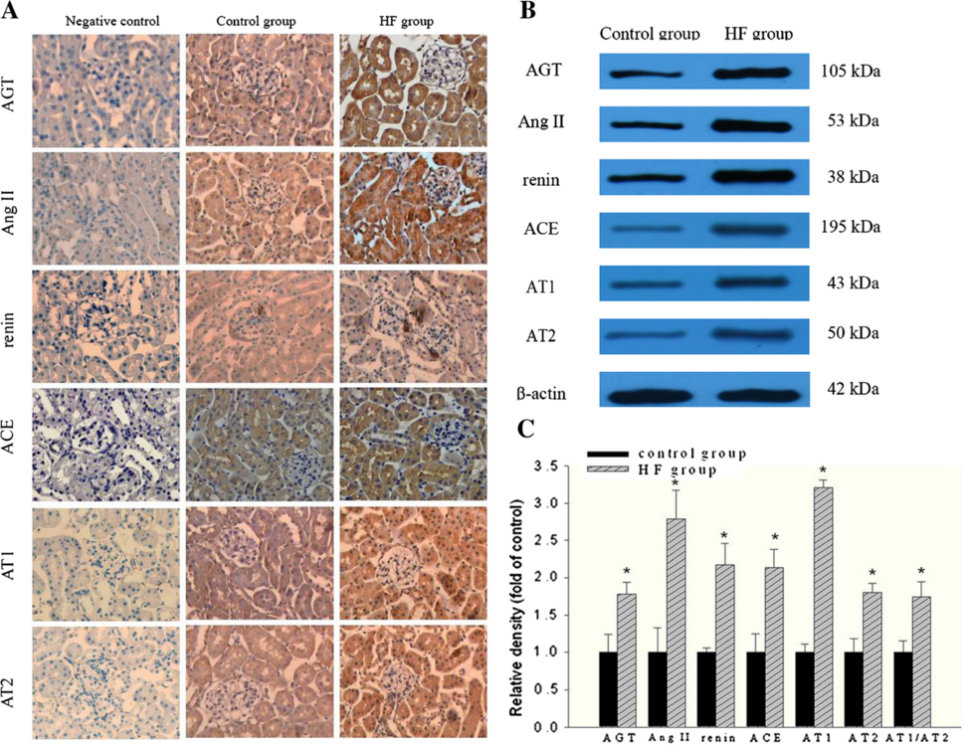
**Protein expressions of intrarenal RAS components in two groups of mice.** (**A**) Immunohistochemical analysis of intrarenal RAS expression position in the two groups of mice. AGT immunoreactivity in the proximal tubular cells, Ang II immunoreactivity in both glomerular and tubular cells, renin immunoreactivity in juxtaglomerular apparatus cells, ACE immunoreactivity in brush border membranes of proximal tubules as well as AT1 and AT2 immunoreactivity in the proximal tubules were increased in the HF group when compared to the control group. (**B**) Western blot analysis for protein expressions of intrarenal RAS components in the two groups of mice. (**C**) The histogram represents mean ± SD of the densitometric scans for the protein bands of angiotensinogen (AGT), angiotensin II (Ang II), renin, angiotensin converting enzyme (ACE), angiotensin II type 1 receptor (AT1), angiotensin II type 2 receptor (AT2) from five experiments, normalized by β-actin. * *P* < 0.05 *vs.* control.

To observe the effects of hyperlipidemia on intrarenal RAS activation and the specific location of RAS expression, we examined the protein expressions of intrarenal RAS components in ApoE KO mice by immunohistochemical staining and Western blot. There were increased protein expressions of angiotensinogen predominantly in the proximal tubular cells, angiotensin II in both glomerular and tubular cells, renin in juxtaglomerular apparatus cells, ACE mainly in brush border membranes of proximal tubules, and AT1 and AT2 in glomeruli and proximal tubules in the HF group when compared to the control group (Figure 3A). These results were further confirmed by Western blot analysis. The protein expressions of all RAS components (angiotensinogen, angiotensin II, renin, ACE, AT1 and AT2) in the kidneys of HF group were increased compared with those of the control group (Figure 3B and 3C). These findings suggest that hyperlipidemia activates intrarenal RAS, especially in renal tubular cells.

### Correlation analysis between plasma lipid profile and intrarenal RAS activation

**Figure 4:**
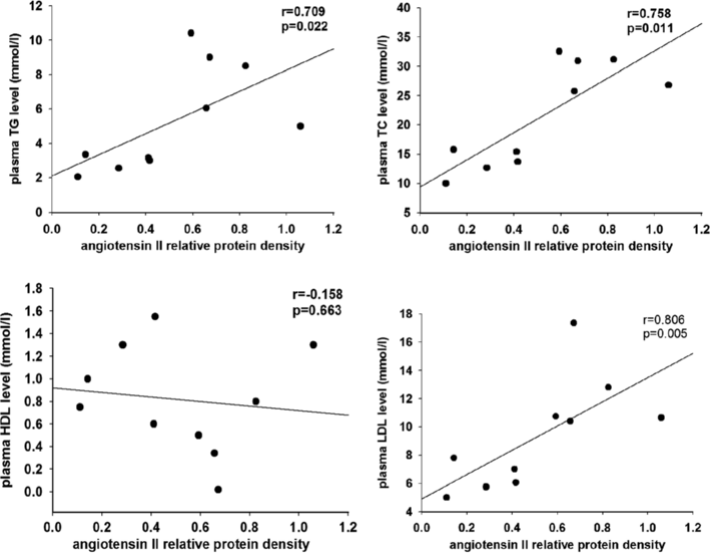
**Correlation analysis between plasma lipid profile and intrarenal RAS activation.** Data from the two groups were compared using the Spearman correlation coefficient. The plasma levels of total cholesterol (TC), triglyceride (TG), and low density lipoprotein (LDL) showed a significant positive correlation with intrarenal angiotensin II expression. *P*-values were two-tailed, and *P* < 0.05 was considered significant.

We analysed the correlation between hyperlipidemia and intrarenal RAS activation analyzed by Western blot (each group n = 5) (Figure 4). A positive correlation was observed between intrarenal angiotensin II expression and plasma levels of TC (r = 0.709, *P* < 0.05), TG (r = 0.758, *P* < 0.05) and LDL (r = 0.806, *P* < 0.05), while no correlation was observed between angiotensin II expression and plasma HDL level (r = −0.158, *P* = 0.663).

### Effects of hyperlipidemia on protein expressions of E-cadherin and α-SMA in kidneys of ApoE KO mice

**Figure 5:**
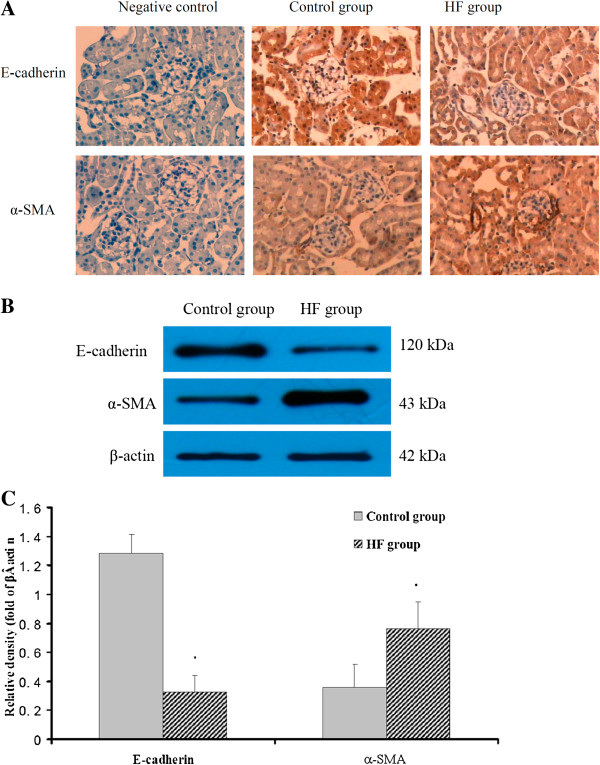
**Effects of hyperlipidemia on protein expressions of α-SMA and E-cadherin (A) Immunohistochemical staining of α-SMA and E-cadherin in kidneys.** Increased expression of α-SMA and decreased expression E-cadherin were observed in the tubular interstitium of the HF group when compared with the control group. (**B**) Western blot analysis for the protein expressions of α-SMA and E-cadherin in kidneys. (**C**) The histogram represents mean ± SD of the densitometric scans for the protein bands of α-SMA and E-cadherin from eight experiments, normalized by comparison with β-actin. * *P* < 0.05 *vs.* control.

We examined the protein expressions of E-cadherin and α-SMA, which are the main biomarkers for the evaluation of EMT, in kidneys of ApoE KO mice. There was increased α-SMA protein expression and decreased E-cadherin protein expression in the renal tubular cells of the HF group compared with those of the control group, as confirmed by immunohistochemical staining (Figure 5A). Western blot analysis reconfirmed the results from the immunohistochemical staining (Figure 5B and 5C). These data strongly suggested that renal tubular cells in the HF group underwent EMT, which might have contributed to the progression of tubular interstitial fibrosis.

### Effects of lipid loading on the expressions of RAS components in HK-2 cells

**Figure 6:**
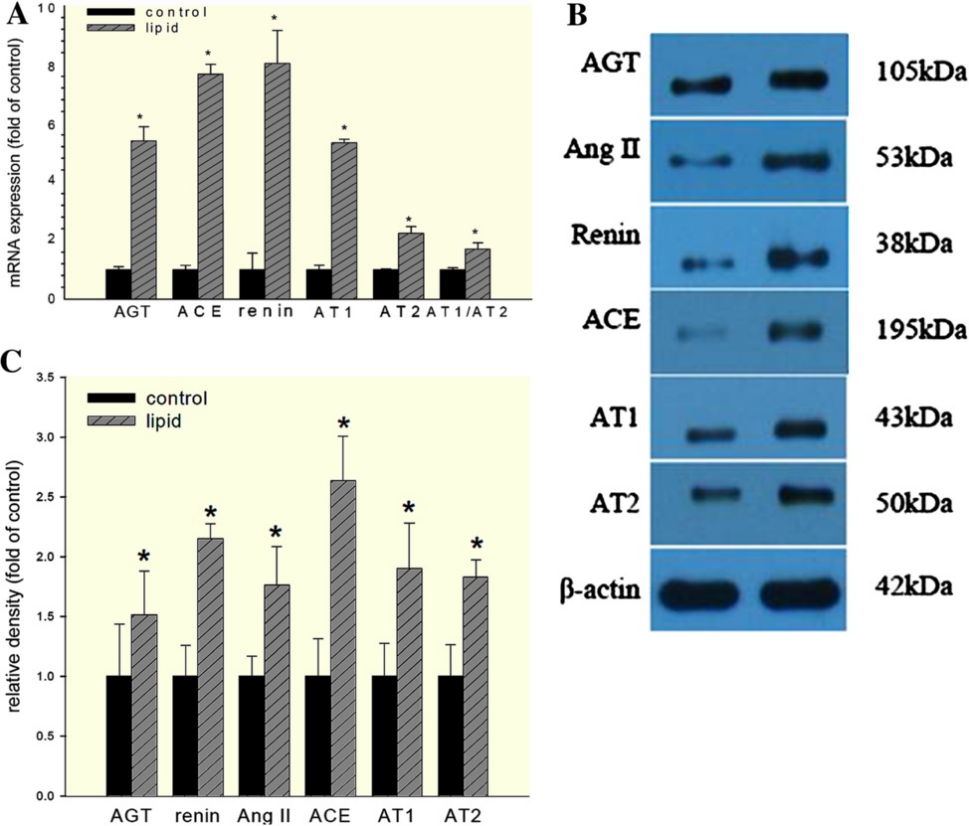
**Up-regulation of RAS induced by cholesterol and 25-hydroxycholesterol in HK-2 cells.** HK-2 cells were made quiescent by serum-free medium for 24 hours and then maintained in serum-free medium (control) or serum-free medium containing 30 μg/ml cholesterol together with 1 μg/ml 25-hydroxycholesterol (lipid) for 24 hours. (**A**) Real-time PCR of the total RAS components mRNA prepared from HK-2 cells with or without lipid treatment. AT1/AT2 ratio was evaluated as the balance between AT1 and AT2.β-actin was used as mRNA loading control. (**B**) Western blot analysis for RAS components protein expression. (**C**) The histogram shows the average volume density corrected by the housekeeping control, β-actin. Data is expressed as mean ± SD. *P < 0.05 vs. control. Angiotensinogen, AGT; angiotensin II, Ang II; angiotensin converting enzyme, ACE; angiotensin II type 1 receptor, AT1; angiotensin II type 2 receptor, AT2.

Next, we investigated whether lipid modulate RAS in HK-2 cells by analyzing the effects of lipid loading on the expressions of RAS components in HK-2 cells. The media containing with 30 μg/ml cholesterol and 1 μg/ml 25-hydroxycholesterol were loaded for 24 hours in HK-2 cells. As shown in Figure 6A, there were significant increased intracellular mRNA levels of angiotensinogen, renin, ACE, AT1 and AT2 in HK-2 cells. The balance between AT1 and AT2, presented as AT1/AT2 ratio, was also found increased under the stimulation of cholesterol and 25-hydroxycholesterol. These results were confirmed at the protein level examined by Western blot analysis (Figure 6B).

### HK-2 Cells undergo EMT under cholesterol and 25-hydroxycholesterol loading

**Figure 7:**
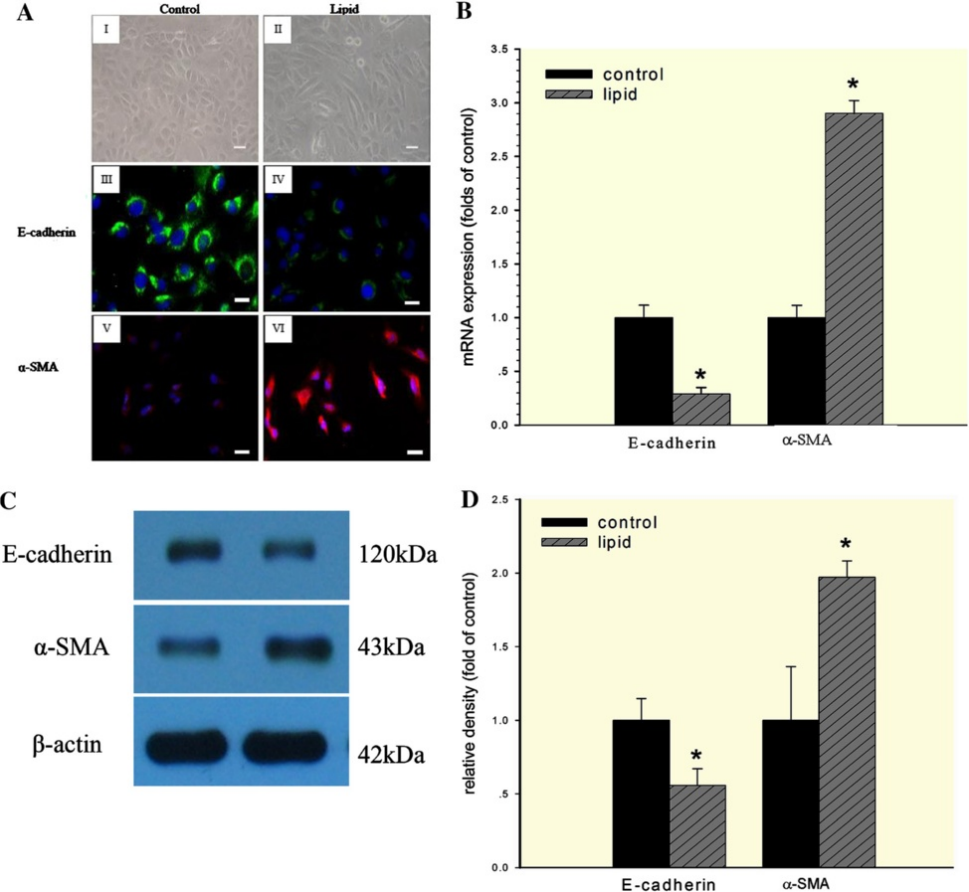
**Lipid loading induced EMT in HK-2 cells.** HK-2 cells were made quiescent by serum-free medium for 24 hours and then maintained in serum-free medium (control) or serum-free medium containing 30 μg/ml cholesterol together with 1 μg/ml 25-hydroxycholesterol (lipid) for 24 hours. (**A**) Morphological changes of HK-2 cells under phase contrast microscopy (I and II, Scale bar: 50 μm) and immunofluorescence analysis of E-cadherin (III and IV, Scale bar: 25 μm) and α-SMA (V and VI, Scale bar: 25 μm) expression in cells with or without lipid treatment. (**B**) Real-time PCR for E-cadherin and α-SMA mRNA expression in HK-2 cells with or without lipid treatment. β-actin was used as mRNA loading control. (**C** and **D**) Western blot analysis for E-cadherin and α-SMA protein expression. The histogram shows the average volume density corrected by housekeeping control, β-actin. Data are expressed as mean ± SD. *P < 0.05 *vs*. control. All of the data shown in these studies are representative of at least three separate experiments.

HK-2 cells were loaded with 30 μg/ml cholesterol together with 1 μg/ml 25-hydroxycholesterol for 24 hours to investigate the lipid effects on EMT. Normal HK-2 cells presented ovoid or cubic morphologies, forming an epithelial monolayer with evidence of a tight cell-cell junction (Figure 7A, I). After treatment with cholesterol and 25-hydroxycholesterol, cells showed notable elongation consistent with the morphology of myofibroblasts (Figure 7A, II). Immunofluoresence analysis showed that HK-2 cells in control group had typical epithelial characteristics of high E-cadherin protein expression and low α-SMA expression (Figure 7A, III and V). After twenty-four- hours incubation with cholesterol and 25-hydroxycholesterol, the cells had a significant reduction in E-cadherin expression and corresponding increase in α-SMA expression (Figure 7A, IV and VI). The mRNA and protein expression of these markers were consistent with the immunofluoresence results (Figure 7B, 7C, and 7D).

## Discussion

Since the proposal of the “lipid nephrotoxicity hypothesis” in 1982 [18], increasing evidence from clinical and experimental studies has supported the hypothesis that lipid abnormalities in CKD lead to progressive renal injury, manifested as resultant glomerulosclerosis and interstitial fibrosis [19]. Lipid deposition, mononuclear cell infiltration and accumulation of extracellular matrix (ECM) components are recognized as early events in the development of glomerulosclerosis, which mimics some of the characteristics of the atherosclerotic vessel wall [20]. Recently, studies have demonstrated that local RAS activation, especially angiotensin II, is implicated in the pathobiology of hypercholesterolemic atherosclerosis [21]. Therefore, in this study, we observed the possible roles of local RAS activation in dyslipidemia mediated renal injury using ApoE KO mouse.

ApoE KO mouse is a classical and commercial model widely used in the research of atherosclerosis and hyperlipidemia-induced peripheral organs injury. ApoE KO mouse is the first model without dietary intervention that shows more severe and more rapid development of atherosclerotic plaques in a model of renal damage [22]. In addition, diets containing with high fat and cholesterol markedly accelerate plaque development in these mice [23]. Our previous studies have demonstrated that high fat diet (Western diet) fed with ApoE KO mice for 8 weeks can induce more severe hyperlipidemia and lipid droplet accumulation in liver and heart [24,25].

Results showed that hyperlipidemia contributed to the development of tubular interstitial fibrosis through EMT, which was closely correlated with increased expressions of local RAS components. These were confirmed by results from *in vitro* study. Lipid loading induced HK-2 cells underwent EMT through intracellular RAS activation. It has been accepted that phenotypic alteration of kidney cells (glomerular endothelial cells, podocytes, tubular epithelial cells, etc.) leads to functional impairment and the progression of CKD [1]. EMT, a process by which differentiated epithelial cells undergo a phenotypic conversion that gives rise to matrix-producing fibroblasts and myofibroblasts, is increasingly recognized as an integral part of tubular interstitial fibrosis after injury [26,27]. We demonstrated that hyperlipidemia induced EMT in the kidneys of HF group and in HK-2 cells, which could be closely associated with ECM deposition in the tubular interstitium.

Among the potential mediators inducing EMT, the RAS is widely acknowledged to play a central role. Our previous studies demonstrated that RAS activation and inflammation contributes to the development of renal interstitial fibrosis and cardiac fibrosis via EMT [28-30]. To evaluate the RAS activation in hyperlipidemia mediated renal injury *in vivo*, we examined the plasma levels and tissue expressions of RAS components in the kidneys. The results showed that expressions of RAS components in the kidneys were markedly upregulated in the HF group compared with the control, suggesting that local RAS, not circulating RAS, may play a more important role in the progression of tissue injuries. Oliveira *et al. *[31] confirmed in Wistar rat models that the local RAS could regulate left ventricular hypertrophy induced by swimming training, independently of circulating renin, which was in accordance with our results. A possible explanation for our circulating RAS results is that the circulating RAS is considered an endocrine axis. An increase in RAS components, such as the angiotensin receptors, may decrease the concentration of other RAS associated molecules via feedback mechanism [32]. In addition, a possible explanation for the reduction of plasma prorenin levels in the HF group is that prorenin was activated without proteolysis by the binding of the prorenin receptor [33], which contributed to the progression of glomerulosclerosis [34]. This suggests that circulating prorenin may bind to the local prorenin receptor, as a part of local RAS, resulting in decreased levels of prorenin in plasma. Correlation analysis between plasma levels of the lipids with the expression of intrarenal angiotensin II further indicated that hyperlipidemia may induce renal injuries partly through intrarenal RAS activation.

Since the immunohischemistry staining showed that the morphological changes and intrarenal RAS activation in kidneys of ApoE KO mice were mainly focused on tubular cells, we used HK-2 cells to further observe the role of lipid on RAS activation. The results revealed that lipid loading caused a significant up-regulation of intracellular RAS components in mRNA and protein levels. These data were in accordance with our *in vivo* results and increasing number of studies supporting the contention that the cholesterol metabolites are regulators of the RAS activation by certain mechanism. Early studies proved an ability of LDL cholesterol to increase AT1 gene expression on vascular smooth muscle cells [35] and later that oxidized LDL can also increase AT1 expression in human coronary artery endothelial cells [36]. Interestingly, both AT1 and AT2 expressions in kidneys of ApoE KO mice and in HK-2 cells were increased after high lipid stimulation in this study. Traditionally, Ang II, the key component of the RAS, acts through AT1 and AT2 receptors. The AT1 regulates the expression of profibrotic factors in kidney diseases, while the AT2 has been thought to counteract the effects of AT1 and to play a role in the protection of the kidney [37]. Recently, the AT2 has also been demonstrated to be involved in some important renal pathophysiological processes. Wolf *et al*. [38] demonstrated in various cell lines and *in vivo* that AT2 through activation of nuclear factor-κB participated in renal inflammatory cell recruitment, and that potential Ang II-mediated proinflammatory effects may not be totally antagonized by the currently increased clinical use of AT1 receptor antagonists, suggesting the balance between AT1 and AT2 may be more valuable in evaluating the kidney injury [39]. In this study, we found that AT1/AT2 ratio was increased *in vivo* and *in vitro*, suggesting that the balance between the two receptors was disrupted.

Our results and those from other studies suggest that the effects of local RAS activity and hyperlipidemia are not independent and that hyperlipidemia enhances local RAS activity. Gross *et al.*[40] confirmed that AT1 expression was upregulated in human atherosclerotic tissues. An *in vitro* study also showed that oxidized LDL upregulated AT1 expression in cultured human coronary artery endothelial cells via the activation of the nuclear transcription factor kappa B (NF-κB) pathway [36]. Tian *et al. *[41] treated *db/db* mice with rosuvastatin and observed that the vasoprotective effects of rosuvastatin are achieved by its inhibition of reactive oxygen species production from the AT1R-NAD(P)H oxidase cascade. Nevertheless, much about the detailed molecular mechanisms is unknown and need to be elucidated.

In summary, our study *in vivo* and *in vitro* proved novel evidence that the activation of local RAS was involved in the hyperlipidemia-mediated renal tubular injuries by inducing EMT of renal tubular cells, thereby accelerating ECM deposition in the tubular interstitium. The potential synergistic effects between hyperlipidemia and RAS activation in the progression of CKD may provide a therapeutic implication that management of blood pressure, dyslipidemia, and proteinuria combined by RAS blockade and statins are not independent components of the treatment regimen.

## Materials and methods

### Ethics statement

This study was carried out in strict accordance with the recommendations in the Guide for the Care and Use of Laboratory Animals of the National Institutes of Health. The protocol was approved by the Committee on the Ethics of Animal Experiments of Southeast University. All surgery was performed under sodium pentobarbital anesthesia, and all efforts were made to minimize suffering.

### Animals

Male apolipoprotein E knockout (ApoE KO) mice with a C57BL/6 genetic background were provided by Animal Care of Chong Qing Medical University. The mice were maintained under a constant 12-hours photoperiod at temperatures between 21°C and 23°C and allowed free access to food and water. Eight-week-old ApoE KO mice fed (randomly assigned) either a normal diet containing 4% fat (control group, n = 8) or a high fat diet (HF group, n = 8) containing 21% fat and 0.15% cholesterol for 8 weeks. At the end of the experimental period, blood samples were obtained for biochemical assays, and kidney samples were used for histological assessments.

### Plasma lipid profile analysis

At termination, the mice were euthanized and blood samples were obtained from the right ventricle for biochemical analysis. Serum concentrations of triglyceride (TG), total cholesterol (TC), high-density lipoprotein (HDL) and low-density lipoprotein (LDL) were determined by automatic analyzers (Hitachi, Japan).

### Determination of angiotensin I, angiotensin II, renin and prorenin in plasma

Plasma concentrations of angiotensinogen, angiotensin I and angiotensin II were determined using a radioimmunoassay kit (Beijing North Institute of Biological Technology, China). Plasma active renin concentrations were assessed by measuring their ability to generate angiotensin I from angiotensinogen. Plasma renin levels were determined by incubating mouse plasma (10 μL diluted in water, 250 μL final volume) with 250 μL of nephrectomized rat plasma containing angiotensinogen levels equivalent to 5000 pg of angiotensin I. The generated angiotensin I was then quantified by radioimmunoassay. The results were expressed as picograms of angiotensin I per milliliter of plasma per hour of incubation. Prorenin concentrations were determined by measuring active renin levels before and after treating plasma with bovine pancreatic trypsin (Invitrogen, USA) at a concentration determined to yield maximum renin activation. The prorenin level for each sample was calculated using the total trypsin-activated renin minus the active rennin [42].

### Cell culture

Human renal proximal tubular epithelial cell line (HK-2) cells immortalized by transduction with human papilloma virus 16 E6/E7 genes were cultured in DMEM/F12 (1:1) (Gibco, USA) culture medium containing 1% penicillin and streptomycin (Invitrogen, USA), 2 mmol/L L-glutamine (Sigma, USA), and 10% heat-inactivated fetal calf serum (Gibco, USA). Cell cultures were maintained in an incubator with a 5% CO_2_ atmosphere at 37°C. At 70-80% confluence, cells were synchronized with serum-free culture medium containing 0.2% fatty acid-free bovine serum albumin (BSA, Gibco, USA) for 24 hours and subsequently stimulated with 30 μg/ml cholesterol (Sigma, USA) plus 1 μg/ml 25-hydroxycholesterol (Sigma, USA) for 24 hours.

### Morphological analysis

Kidney sections were fixed with 4% buffered paraformaldehyde for 24 hours, washed with 70% ethanol for 24 hours and then embedded in paraffin. Four-micrometer-thick sections were prepared for Masson staining and were then examined under light microscopy. To evaluate the extent of glomerulosclerosis and renal interstitial fibrosis, the renal cortex fraction occupied by fibrotic tissue that stained positively for collagen was quantitatively evaluated in Masson-stained sections using Image J software (Version 1.44). We used a point-counting technique in consecutive microscopic fields at a final magnification of ×200 under a 176-point grid.

### Immunohistochemical analysis and immunofluorescence

Localization of RAS components in mice kidney was performed in paraffin-embedded sections. The slides were previously deparaffinized and treated with 0.3% endogenous peroxidase blocking solution for 15 minutes. Sections were then treated sequentially with normal nonimmune animal serum for 30 minutes and incubated with anti-mouse polyclonal primary antibodies of angiotensinogen (diluted 1:500, Abbiotec, USA), renin (diluted 1:200, Santa Cruz Biotechnology Inc.,USA), angiotensin II (diluted 1:400, Novus Biologicals, USA), ACE (diluted 1:200, Santa Cruz Biotechnology Inc., USA), AT1 (diluted 1:200, Santa Cruz Biotechnology Inc., USA), AT2 (diluted 1:200, Santa Cruz Biotechnology Inc., USA), E-cadherin (diluted 1:200, Santa Cruz Biotechnology Inc., USA) and α-smooth muscle actin (α-SMA, diluted 1:400, Abcam, UK) at 4°C overnight. Sections were then incubated with biotin-labeled secondary antibodies (Maixin Biotechnology Ltd., China) for 30 minutes at room temperature, followed by incubation with streptomycete antibiotin-peroxidase for another 10 minutes. Staining was completed by 3-minute incubation with 3, 3^′^-diaminobenzidine substrate-chromogen, which resulted in a brown-colored precipitate at the antigen site. Counterstaining was performed with hematoxylin. Immunohistochemical images were acquired by light microscopy.

For immunocytochemical analysis, HK-2 cells were cultured on sterile glass coverslips in 24-well plates. Thereafter, cultures were treated and fixed with iced paraformaldehyde for 10 minutes, permeabilized with 0.25% Triton X-100 for 10 minutes, and blocked with 5% BSA at 37°C for an additional 30 minutes. The cells were then incubated with primary anti-E-cadherin and anti-α-SMA antibodies overnight at 4°C, followed by incubation with Alexa Fluor labeled secondary antibody at 37°C for 1 hours. Finally, slides were examined with an Olympus DP71 fluorescence microscope.

### Real-time polymerase chain reaction (real time-PCR)

Total RNA was extracted from HK-2 cells by RNAiso plus reagent and reverse transcription was performed using the standard reagent (Takara, Japan) in accordance with the protocols. Real time-PCR was performed on ABI PRISM 7300 real-time PCR System (Applied Biosystems, USA) using SYBR Green dye. The primers used for real-time PCR were given in Table 2. β-actin served as internal reference gene. Results were analyzed using Sequence Detection Software version 1.4 (Applied Biosystems, USA). The relative gene expression of each target was quantified against a standard curve. The pre-PCR product of each gene was used as standard, and the standard curve was established with a 10-fold serial dilution of the product. The standard curve was included in all PCR runs. The equation of target gene abundance/reference gene abundance was used to evaluate the level of expression of each gene. All measurements were performed in duplicate.

**Table 2:** Primers for real-time polymerase chain reaction

**Gene**	**Primer sequences**
E-cadherin	5^′^-AAATCTGAAAGCGGCTGATACTG-3^′^-sense
	5^′^-CGGAACCGCTTCCTTCATAG-3^′^-antisense
α-SMA	5^′^-GACAATGGCTCTGGGCTCTGTAA-3^′^-sense
	5^′^-ATGCCATGTTCTATCGGGTACTTCA-3^′^-antisense
angiotensinogen	5^′^-GATGTTGCTGCTGAGAAGATTG-3^′^-sense
	5^′^-GGAAGTGGACGTAGGTGTTGA-3^′^-antisense
renin	5^′^-GAGGCTGACACTTGGCAACA-3^′^-sense
	5^′^-CGCCATAGTACTGGGTGTCCAT-3^′^-antisense
ACE	5^′^-CACTATCAAGCGGATCATAAAGAAG-3^′^-sense
	5^′^-CACGCTGTAGGTGGTTTCCATA-3^′^-antisense
AT1	5^′^-ACCTGGCTATTGTTCACCCAAT-3^′^-sense
	5^′^-TGCAGGTGACTTTGGCTACAAG-3^′^-antisense
AT2	5^′^-CCACCCTTGCCACTACTAGCA-3^′^-sense
	5^′^-ATTGTTGCCAGAGATGTTCACAA-3^′^-antisense
β-actin	5^′^-AAAGACCTGTACGCCAACAC-3^′^-sense
	5^′^-GTCATACTCCTGCTTGCTGAT-3^′^-antisense

### Western blot analysis

The total proteins from kidney homogenates of ApoE KO mice or cell extracts were separated by sodium dodecyl sulfate polyacrylamide gel electrophoresis and then transferred to a nitrocellulose membrane. After blocking with 5% skim milk in Tris-buffered saline with 0.5% Tween 20 overnight at 4°C, the membrane was incubated with the primary antibodies of angiotensinogen, angiotensin II, renin, ACE, AT1, AT2, E-cadherin and α-SMA for 1 hour at room temperature, followed by incubation with the appropriate horseradish peroxidase-conjugated secondary antibodies for another one hour. Finally, the signals were detected using an ECL Advanced™ system (GE Healthcare, USA). Relative protein levels were calculated by normalization to the amount of β-actin protein.

### Statistical analysis

All data are presented as the mean ± SD. Comparisons between different two groups were performed using an unpaired Student’s *t* test before expressing the results as a percentage of the control value. The correlation between the plasma lipid profile and the protein expression of angiotensin II in kidneys of ApoE KO mice were calculated with the Spearman rank-order correlation using SPSS 13.0 software. A *P* value less than 0.05 was considered significant.

## Abbreviations

RAS: Renin-angiotensin system; CKD: Chronic kidney disease; HF: High fat; HK-2: Human proximal tubular epithelial cell line; EMT: Epithelial mesenchymal transition; TIF: Tubular interstitial fibrosis; TG: Triglyceride; TC: Total cholesterol; VLDL: Very-low-density lipoprotein; LDL: Low-density lipoprotein; HDL: High-density lipoprotein cholesterol; GFR: Glomerular filtration rate; AT1: Angiotensin II type 1 receptor; AT2: Angiotensin II type 2 receptor; ACE: Angiotensin-converting enzyme; ApoE KO: Apolipoprotein E knockout; ECM: Extracellular matrix; NF-κB: Nuclear transcription factor kappa B.

## Competing interests

The authors declare that they have no competing interests.

## Authors’ contributions

Design of the study: MKL, RXZ, and LBC; Conduct of the study: NJ, LJ, LLL, and NHF; data collection: WCX; data analysis: NJ and MKL; manuscript writing: NJ, MKL, and LBC; Final approval: NJ, M-KL, W-CX, LJ, ZY, L-LL, N-HF, C-YX, R-XZ, and L-BC. All authors read and approved the final manuscript.

## References

[B1] Liu Y New insights into epithelial-mesenchymal transition in kidney fibrosis J Am Soc Nephrol 2010 21 2 212 222 20019167 10.1681/ASN.2008121226 PMC4554339

[B2] Rodriguez-Iturbe B Johnson RJ Herrera-Acosta J Tubulointerstitial damage and progression of renal failure Kidney Int Suppl 2005 99 S82 S86 16336583 10.1111/j.1523-1755.2005.09915.x

[B3] Chauhan V Vaid M Dyslipidemia in chronic kidney disease: managing a high-risk combination Postgrad Med 2009 121 6 54 61 19940417 10.3810/pgm.2009.11.2077

[B4] Diepeveen SHA Wetzels JFM Bilo HJG van Tits LJH Stalenhoef AFH Cholesterol in end-stage renal disease: the good, the bad or the ugly? Neth J Med 2008 66 2 53 61 18292607

[B5] Kwan BC Kronenberg F Beddhu S Cheung AK Lipoprotein metabolism and lipid management in chronic kidney disease J Am Soc Nephrol 2007 18 4 1246 1261 17360943 10.1681/ASN.2006091006

[B6] Kobori H Nangaku M Navar LG Nishiyama A The intrarenal renin-angiotensin system: from physiology to the pathobiology of hypertension and kidney disease Pharmacol Rev 2007 59 3 251 287 17878513 10.1124/pr.59.3.3

[B7] Fogo AB The role of angiotensin II and plasminogen activator inhibitor-1 in progressive glomerulosclerosis Am J Kidney Dis 2000 35 2 179 188 10676714 10.1016/s0272-6386(00)70324-6

[B8] Ruster C Wolf G Renin-angiotensin-aldosterone system and progression of renal disease J Am Soc Nephrol 2006 17 11 2985 2991 17035613 10.1681/ASN.2006040356

[B9] Wolf G Renal injury due to renin-angiotensin-aldosterone system activation of the transforming growth factor-beta pathway Kidney Int 2006 70 11 1914 1919 16985515 10.1038/sj.ki.5001846

[B10] Dzau VJ Re R Tissue angiotensin system in cardiovascular medicine. A paradigm shift? Circulation 1994 89 1 493 498 8281685 10.1161/01.cir.89.1.493

[B11] Kumar R Boim MA Diversity of pathways for intracellular angiotensin II synthesis Curr Opin Nephrol Hypertens 2009 18 1 33 39 19077687 10.1097/MNH.0b013e32831a9e20

[B12] Hamden K Keskes H Belhaj S Mnafgui K Feki A Allouche N Inhibitory potential of omega-3 fatty and fenugreek essential oil on key enzymes of carbohydrate-digestion and hypertension in diabetes rats Lipids Health Dis 2011 10 226 22142357 10.1186/1476-511X-10-226 PMC3240899

[B13] Singh BK Mehta JL Interactions between the renin-angiotensin system and dyslipidemia: relevance in atherogenesis and therapy of coronary heart disease Indian Heart J 2001 53 4 511 518 11759948

[B14] Catar RA Muller G Heidler J Schmitz G Bornstein SR Morawietz H Low-density lipoproteins induce the renin-angiotensin system and their receptors in human endothelial cells Horm Metab Res 2007 39 11 801 805 17992634 10.1055/s-2007-991158

[B15] Luo P Yan M Frohlich ED Mehta JL Hu C Novel concepts in the genesis of hypertension: role of LOX-1 Cardiovasc Drugs Ther 2011 25 5 441 449 21912849 10.1007/s10557-011-6337-1

[B16] Lu J Mehta JL LOX-1: a critical player in the genesis and progression of myocardial ischemia Cardiovasc Drugs Ther 2011 25 5 431 440 21847544 10.1007/s10557-011-6329-1

[B17] Wang X Phillips MI Mehta JL LOX-1 and angiotensin receptors, and their interplay Cardiovasc Drugs Ther 2011 25 5 401 417 21861069 10.1007/s10557-011-6331-7 PMC7102029

[B18] Moorhead JF Chan MK El-Nahas M Varghese Z Lipid nephrotoxicity in chronic progressive glomerular and tubulo-interstitial disease Lancet 1982 2 8311 1309 1311 6128601 10.1016/s0140-6736(82)91513-6

[B19] Trevisan R Dodesini AR Lepore G Lipids and renal disease J Am Soc Nephrol 2006 17 4 Suppl 2 S145 S147 16565240 10.1681/ASN.2005121320

[B20] Wheeler DC Chana RS Interactions between lipoproteins, glomerular cells and matrix Miner Electrolyte Metab 1993 19 3 149 164 8232102

[B21] Chen J Li D Schaefer R Mehta JL Cross-talk between dyslipidemia and renin-angiotensin system and the role of LOX-1 and MAPK in atherogenesis studies with the combined use of rosuvastatin and candesartan Atherosclerosis 2006 184 2 295 301 16005008 10.1016/j.atherosclerosis.2005.04.016

[B22] Buzello M Tornig J Faulhaber J Ehmke H Ritz E Amann K The apolipoprotein e knockout mouse: a model documenting accelerated atherogenesis in uremia J Am Soc Nephrol 2003 14 2 311 316 12538731 10.1097/01.asn.0000045048.71975.fc

[B23] Pendse AA rbones-Mainar JM Johnson LA Altenburg MK Maeda N Apolipoprotein E knock-out and knock-in mice: atherosclerosis, metabolic syndrome, and beyond J Lipid Res 2009 50 Suppl S178 S182 19060252 10.1194/jlr.R800070-JLR200 PMC2674752

[B24] Ma KL Ruan XZ Powis SH Chen Y Moorhead JF Varghese Z Inflammatory stress exacerbates lipid accumulation in hepatic cells and fatty livers of apolipoprotein E knockout mice Hepatology 2008 48 3 770 781 18752326 10.1002/hep.22423

[B25] Ma KL Liu J Ni J Zhang Y Lv LL Tang RN Ni HF Ruan XZ Liu BC Inflammatory stress exacerbates the progression of cardiac fibrosis in high-fat-fed apolipoprotein E knockout mice via endothelial-mesenchymal transition Int J Med Sci 2013 10 4 420 426 23471419 10.7150/ijms.5723 PMC3590602

[B26] Acloque H Adams MS Fishwick K Bronner-Fraser M Nieto MA Epithelial-mesenchymal transitions: the importance of changing cell state in development and disease J Clin Invest 2009 119 6 1438 1449 19487820 10.1172/JCI38019 PMC2689100

[B27] Thiery JP Acloque H Huang RY Nieto MA Epithelial-mesenchymal transitions in development and disease Cell 2009 139 5 871 890 19945376 10.1016/j.cell.2009.11.007

[B28] Dai HY Zheng M Tang RN Ni J Ma KL Li Q Liu BC Effects of angiotensin receptor blocker on phenotypic alterations of podocytes in early diabetic nephropathy Am J Med Sci 2011 341 3 207 214 21326079 10.1097/MAJ.0b013e3182010da9

[B29] Li Q Liu BC Lv LL Ma KL Zhang XL Phillips AO Monocytes induce proximal tubular epithelial-mesenchymal transition through NF-kappa B dependent upregulation of ICAM-1 J Cell Biochem 2011 112 6 1585 1592 21344487 10.1002/jcb.23074

[B30] Tang RN Lv LL Zhang JD Dai HY Li Q Zheng M Ni J Ma KL Liu BC Effects of angiotensin II receptor blocker on myocardial endothelial-to-mesenchymal transition in diabetic rats Int J Cardiol 2013 162 2 92 99 21704391 10.1016/j.ijcard.2011.06.052

[B31] Oliveira EM Sasaki MS Cerencio M Barauna VG Krieger JE Local renin-angiotensin system regulates left ventricular hypertrophy induced by swimming training independent of circulating renin: a pharmacological study J Renin Angiotensin Aldosterone Syst 2009 10 1 15 23 19286754 10.1177/1470320309102304

[B32] Schefe JH Menk M Reinemund J Effertz K Hobbs RM Pandolfi PP Ruiz P Unger T Funke-Kaiser H A novel signal transduction cascade involving direct physical interaction of the renin/prorenin receptor with the transcription factor promyelocytic zinc finger protein Circ Res 2006 99 12 1355 1366 17082479 10.1161/01.RES.0000251700.00994.0d

[B33] Ichihara A Hayashi M Kaneshiro Y Suzuki F Nakagawa T Tada Y Koura Y Nishiyama A Okada H Uddin MN Nabi AH Ishida Y Inagami T Saruta T Inhibition of diabetic nephropathy by a decoy peptide corresponding to the “handle” region for nonproteolytic activation of prorenin J Clin Invest 2004 114 8 1128 1135 15489960 10.1172/JCI21398 PMC522242

[B34] Ichihara A Suzuki F Nakagawa T Kaneshiro Y Takemitsu T Sakoda M Prorenin receptor blockade inhibits development of glomerulosclerosis in diabetic angiotensin II type 1a receptor-deficient mice J Am Soc Nephrol 2006 17 7 1950 1961 16738017 10.1681/ASN.2006010029

[B35] Nickenig G Sachinidis A Michaelsen F Bohm M Seewald S Vetter H Upregulation of vascular angiotensin II receptor gene expression by low-density lipoprotein in vascular smooth muscle cells Circulation 1997 95 2 473 478 9008466 10.1161/01.cir.95.2.473

[B36] Li D Saldeen T Romeo F Mehta JL Oxidized LDL upregulates angiotensin II type 1 receptor expression in cultured human coronary artery endothelial cells: the potential role of transcription factor NF-kappaB Circulation 2000 102 16 1970 1976 11034947 10.1161/01.cir.102.16.1970

[B37] Wenzel UO Krebs C Benndorf R The angiotensin II type 2 receptor in renal disease J Renin Angiotensin Aldosterone Syst 2010 11 1 37 41 19861345 10.1177/1470320309347787

[B38] Wolf G Wenzel U Burns KD Harris RC Stahl RA Thaiss F Angiotensin II activates nuclear transcription factor-kappaB through AT1 and AT2 receptors Kidney Int 2002 61 6 1986 1995 12028439 10.1046/j.1523-1755.2002.00365.x

[B39] Siragy HM AT1 and AT2 receptor in the kidney: role in health and disease Semin Nephrol 2004 24 2 93 100 15017521 10.1016/j.semnephrol.2003.11.009

[B40] Gross CM Gerbaulet S Quensel C Kramer J Mittelmeier HO Luft FC Dietz R Angiotensin II type 1 receptor expression in human coronary arteries with variable degrees of atherosclerosis Basic Res Cardiol 2002 97 4 327 333 12111043 10.1007/s00395-002-0356-9

[B41] Tian XY Wong WT Xu A Chen ZY Lu Y Liu LM Lee VW Lau CW Yao X Huang Y Rosuvastatin improves endothelial function in db/db mice: role of angiotensin II type 1 receptors and oxidative stress Br J Pharmacol 2011 164 2b 598 606 21486274 10.1111/j.1476-5381.2011.01416.x PMC3188899

[B42] Kantorowicz L Valego NK Tang L Figueroa JP Chappell MC Carey LC Rose JC Plasma and renal renin concentrations in adult sheep after prenatal betamethasone exposure Reprod Sci 2008 15 8 831 838 19017818 10.1177/1933719108318599 PMC2675707

